# A data comparison between a traditional and the single-step β-galactosidase assay

**DOI:** 10.1016/j.dib.2016.05.063

**Published:** 2016-06-01

**Authors:** Jorrit Schaefer, Goran Jovanovic, Ioly Kotta-Loizou, Martin Buck

**Affiliations:** Department of Life Sciences, Faculty of Natural Sciences, Imperial College London, London SW7 2AZ, UK

**Keywords:** LacZ, B-galactosidase, β-Galactosidase, B-Gal, β-Gal, Microplate reader

## Abstract

This article describes reproducibility of a single-step automated β-galactosidase, and the equivalence of its data to the traditional assay (“Experiments in Molecular Genetics” [1]). This was done via a pairwise comparison of both methods using strains with Miller Unit [MU] values ranging from 0 to over 2000. The data presented in this article is associated with the research article entitled “A single-step method for mid to high throughput β-galactosidase assays in *Escherichia coli* using a microplate reader” [2].

## **Specifications Table**

**Table:** 

Subject area	Biology
More specific subject area	Enzymatic assays
Type of data	Table
How data was acquired	FLUOstar Omega Microplate Reader (BMG LABTECH)
Data format	Raw/Analysed
Experimental factors	None applied
Experimental features	β-galactosidase assay
Data source location	Imperial College Road, Imperial College, London SW7 2AZ, UK
Data accessibility	Data is with this article

## **Value of the Data**

• Validates a single-step automated assay to measure β-galactosidase activity in *E. coli* with a microplate reader.
• Establishes the method provides consistent data, with a coefficient of variation similar to that of the traditional assay.
• Student׳s *t*-test demonstrates equivalence of the single-step and the traditional assay data.

## 1 Data

The data presented in this article is a pairwise comparison of the MU activities of the traditional β-galactosidase assay and a single-step automated method [2]. 4 different strains with MU activities (0–10, 40–70, 500–900, and 1800–2400 respectively) were assayed using both methods.

## 2 Experimental design, materials and methods

### 2.1 Cell culture and sample preparation

Samples were grown overnight at 37 °C in LB media. For the traditional assay, samples were treated as described by Miller [1]. For the single-step automated microplate assay, 80 μL of culture was transferred into 96-well Costar® flat bottom microplates (Sigma-Aldrich). 120 μL of a custom mix was added to each well used, including the blank (containing 80 μL LB). The custom mix consists of 60 mM Na_2_HPO_4_, 40 mM NaH_2_PO_4_, 10 mM KCl, 1 mM MgSO_4_, 36 mM β-mercaptoethanol, 166 µL/ml T7 lysozyme, 1.1 mg/ml ONPG, 6.7% PopCulture® Reagent. The Costar microtitre plates were then placed in a FLUOstar Omega Microplate Reader (BMG LABTECH).

### 2.2 Kinetic OD_600_ and OD_420_ measurements

The FLUOstar Omega Microplate Reader was set to 30 °C, with OD_600_ and OD_420_ readings taken every 60 s for 1 h (20 flashes per well each cycle). Wells were set to shake at 500 rpm (double orbital shaking) between readings to ensure homogenisation of the sample and more efficient lysis. A script to run these parameters on a FLUOstar Omega Microplate Reader can be found in Supplementary Data A.

### 2.3 MARS Data Analysis

The MARS Data Analysis software package was used to process the OD_600_ and OD_420_ data generated by the by FLUOstar Omega Microplate Reader. Miller Units values are expressed as follows:

$$\mathrm{Miller}\;\mathrm{Units}=\frac{1000*(\mathrm{OD}420/\min)}{\mathrm{OD}600*\mathrm{volume used}(\mathrm{ml})}=\frac{1000*(\mathrm{OD}420/\min)}{\left(\mathrm{OD}600*2.5\right)*0.080}=\frac{5000*(\mathrm{OD}420/\min)}{\mathrm{OD}600}$$

The kinetic OD_420_ readings were converted into the slope of OD_420_ over time (OD_420_/min), multiplied by 5000, and adjusted for the initial OD_600_ reading at the first time point. The obtained MU values were then compared to those of the traditional assay (Table 1). More detailed lab notes on running this assay and subsequent processing in MARS Data Analysis are available in Supplementary Data B.

### 2.4 Data comparison between the single-step β-galactosidase assay and the traditional assay

See Table 1.

## Figures and Tables

**Table 1:** The single-step automated assay was consistent over multiple orders of magnitude and generated data equivalent to that of the traditional assay. The coefficients of variation of the Miller Unit [MU] measurements using the one-step and the traditional assay were similar for all β-galactosidase expressing strains suggesting that the two methods have comparable precision and reproducibility. Additionally, no statistically significant difference was observed between the single-step and traditional β-galactosidase assay (two-tailed Student׳s *t*-test assuming unequal variance; *P*>0.05).

	**One-step assay**			**Traditional assay**			***T*-test**
	[MU]	Average	Coefficient of variation	[MU]	Average	Coefficient of variation	*P* value
MG1655 Δ*lacZ*	−13	**−2**	–	1.1	**1.1**	–	***0.71***
	12			1.3			
	−5			1			
							
MG1655 Δ*lacZ* ΦP_*pspA*_–*lacZ*	87	**69**	0.23	52	**42**	0.18	***0.12***
	48			35			
	72			37			
							
MG1655 Δ*lacZ* ΦP_*pspA*_–*lacZ* Δ*pspA*	691	**649**	0.05	665	**676**	0.01	***0.35***
	642			677			
	613			686			
							
MC1061 Δ*lacZ* ΦP_*pspA*_–*lacZ ΔpspA*	2985	**2600**	0.11	2070	**2202**	0.09	***0.17***
	2464			2473			
	2351			2062			

## Footnotes

Appendix A Supplementary data associated with this article can be found in the online version at http:dx.doi.org/10.1016/j.dib.2016.05.063.
