# Supplementary material for: A data comparison between a traditional and the single-step β-galactosidase assay
Source: Data Brief. 2016 Jun 1;8:350–2. doi: 10.1016/j.dib.2016.05.063 (PMC4908277; doi:10.1016/j.dib.2016.05.063)
Supplement: Supplementary file 1 — Supplementary material [file mmc1.docx]

Manuscript title:
**A data comparison between a traditional and the single-step β-galactosidase assay**

Authors and affiliations:
**Jorrit Schaefer^1^, Goran Jovanovic^1^, Ioly Kotta-Loizou^1^, Martin Buck^1^**

**^1^Department of Life Sciences, Faculty of Natural Sciences, Imperial College London, London SW7 2AZ, UK**

The authors declare no conflict of interest.
