# Supplementary material for: A data comparison between a traditional and the single-step β-galactosidase assay
Source: Data Brief. 2016 Jun 1;8:350–2. doi: 10.1016/j.dib.2016.05.063 (PMC4908277; doi:10.1016/j.dib.2016.05.063)
Supplement: Supplementary file 3 — Supplementary material [file mmc3.docx]

Protocol for single-step microtitre plate reader β-Gal assay

1. Open the “Single Step Bgal Script.TSC” file (Supplementary Data A) with the FLUOstar Omega Microplate Reader.
2. Select the layout as appropriate. When using more or less than current number of wells the cycle time can be adjusted (under Basic Parameters). The check timing option can be used as a guide.
3. Add 80 μl cells to each well (80 μl LB for blank).
4. Add 120 μl BGal mix to each well with a multichannel pipette, including the blank. Reaction starts now so proceed quickly.
   1. ml BGal mix contains (10 reactions):
      - 800 μl Z buffer with 2.7 µl/ml BME
      - 300 μl Z buffer with 4 mg/ml ONPG
      - 80 μl PopCulture

- 20 μl lysozyme stock solution (10 mg/ml lysozyme in 10mM Tris-HCl pH8 or water)

1. Insert the plate into the plate reader, start measurement and wait for the program to run. Time is currently set for about 1 hour but can be stopped as soon as the OD_420_ is linear for a few minutes. 10-15 minutes is typically sufficient.
2. Next open the data, which will look as follows:


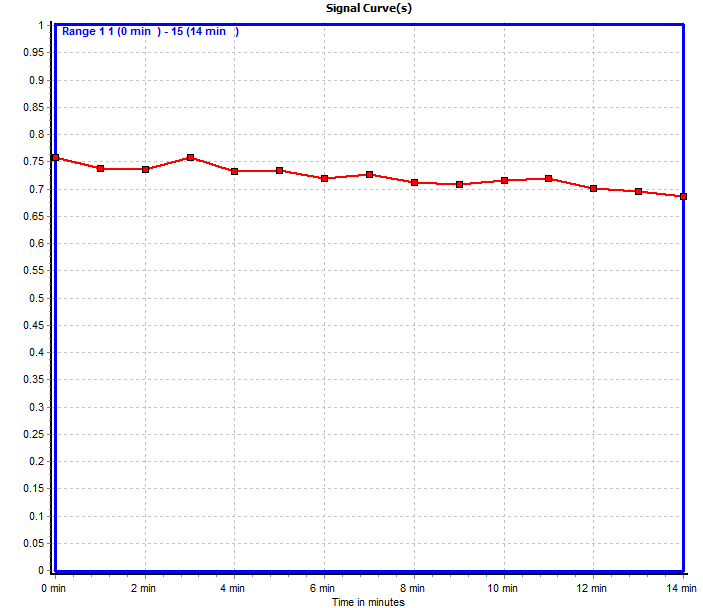
**
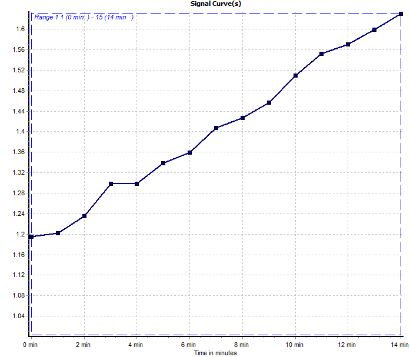
 OD_420_ OD_600_**

1. The OD_420_ should be a linear increase over time, the slope representing the activity per minute. OD_600_ is typically fairly constant; lysis only seems to affect OD_600_ slightly.

$$Miller Units=\frac{1000*(OD420/min)}{Initial culture OD600*volume used (ml)}$$

2. To determine OD_420_/minute, go to Calculations/Kinetics/Kinetic Calculations. Select Blank corrected raw data over the time course of the experiment, select slope and make sure the time is in minutes. If the OD_420_ does not show a linear increase over time for the whole experiment (e.g. ONPG has run out and OD_420_ has reached a plateau), only select the time range where it increases linearly over time.
3. Next go to Calculations/Data Calculations. First input data is Slope of Range 1 based on blank corrected at OD_420_. The multiplier is 5000 (1000 from Miller Unit formula, 12.5 from culture volume and 0.4 from OD_600_ dilution). Divided by second input data: cycle 0 based on blank corrected for OD_600_. All wells now display their respective Miller Units.
